# Sorghum Grains Grading for Food, Feed, and Fuel Using NIR Spectroscopy

**DOI:** 10.3389/fpls.2021.720022

**Published:** 2021-09-16

**Authors:** Irsa Ejaz, Siyang He, Wei Li, Naiyue Hu, Chaochen Tang, Songbo Li, Meng Li, Boubacar Diallo, Guanghui Xie, Kang Yu

**Affiliations:** ^1^College of Agronomy and Biotechnology, China Agricultural University, Beijing, China; ^2^Crops Research Institute, Guangdong Academy of Agricultural Sciences, Guangzhou, China; ^3^College of Bioscience and Biotechnology, Hunan Agricultural University, Changsha, China; ^4^Hunan Branch, National Energy R&D Center for Non-food Biomass, Hunan Agricultural University, Changsha, China; ^5^Department of Agriculture, High Institute Agronomic and Veterinary, Faranah, Guinea; ^6^National Energy R&D Center for Non-food Biomass, China Agricultural University, Beijing, China; ^7^School of Life Sciences, Technical University of Munich, Freising, Germany

**Keywords:** FT-NIR, sorghum grains, biochemical composition, food, feed, fuel, PLSR

## Abstract

Near-infrared spectroscopy (NIR) is a non-destructive, fast, and low-cost method to measure the grain quality of different cereals. However, the feasibility for determining the critical biochemicals, related to the classifications for food, feed, and fuel products are not adequately investigated. Fourier-transform (FT) NIR was applied in this study to determine the eight biochemicals in four types of sorghum samples: hulled grain flours, hull-less grain flours, whole grains, and grain flours. A total of 20 hybrids of sorghum grains were selected from the two locations in China. Followed by FT-NIR spectral and wet-chemically measured biochemical data, partial least squares regression (PLSR) was used to construct the prediction models. The results showed that sorghum grain morphology and sample format affected the prediction of biochemicals. Using NIR data of grain flours generally improved the prediction compared with the use of NIR data of whole grains. In addition, using the spectra of whole grains enabled comparable predictions, which are recommended when a non-destructive and rapid analysis is required. Compared with the hulled grain flours, hull-less grain flours allowed for improved predictions for tannin, cellulose, and hemicellulose using NIR data. This study aimed to provide a reference for the evaluation of sorghum grain biochemicals for food, feed, and fuel without destruction and complex chemical analysis.

## Introduction

Sorghum (*Sorghum bicolor* L.) is the fifth most commanding cereal crop in the world and its grain production has reached up to 57.50 million tons in 2020 (de Morais Cardoso et al., 2017; Food and Agriculture Organization of the United Nations, 2020; Stamenković et al., 2020). Sorghum grain is known for its nutritional quality and there is a worldwide growing market of sorghum grain for use as human food and consumed in the preparation of many foods (Ratnavathi and Patil, 2014; Bader Ul Ain et al., 2019; Sihono et al., 2019; Palacios et al., 2021). It is an excellent feed for animals and its feeding value is generally considered more than 95% of the feeding value of yellow dent maize (Waniska et al., 2016). In addition, it is a unique energy crop that can be used through numerous production routes—for instance, converting starch into ethanol, lignocelluloses into bio-oil, biochar, and biohydrogen, and fat into biodiesel (Mirfakhar et al., 2020; Stamenković et al., 2020). Sorghum cultivars can be classified into four classes: sorghum, white sorghum, tannin sorghum, and mixed sorghum according to the US Grain Standards (Waniska et al., 2016). The grains from different cultivars differ in the presence or absence of thick outer-covering/pericarp called hulled or hull-less grains (Waniska and Rooney, 2000; Guindo et al., 2016). Sorghum for food must have lower fat but higher starch and protein for good digestibility, whereas for feed, it must have higher protein content but lower concentrations of tannin and insoluble dietary fibers especially hemicellulose (Waniska et al., 2016). The insoluble dietary fibers are desirable by many biofuel industries due to its property of making a low viscous solution (Fuller et al., 2016; Qiu et al., 2017; Stamenković et al., 2020). Therefore, a precise classification of sorghum grains requires the determination of various chemical components.

Sorghum grain hulls comprise of condensed tannins that have cross-linkages with starch, protein, and lignocellulosic components (Evers and Millar, 2002; Siwela et al., 2007; Barros et al., 2012; Gyori, 2017). Cellulose, hemicellulose, and lignin are important soluble and insoluble dietary fibers that have complex structural polymerization and are difficult to be determined directly. Unlike other cereal grains, cellulose, hemicellulose, and lignin in sorghum grains are washed out with other sugars during their extraction process (Jin et al., 2017; Li et al., 2017; Krasznai et al., 2018). To cope with this problem, acid hydrolysis has to be done several times, which, however, is not desirable due to specialized laboratory facilities (Ricardo Soccol et al., 2011; Heredia-Olea et al., 2012). In addition, these wet-chemical methods are strenuous, time consuming, and expensive for large samples, making it infeasible for real-time sorting of grains (Orman and Schumann, 1991; Guindo et al., 2016; Yang et al., 2016; Caporaso et al., 2018; Srivastava et al., 2018). Therefore, inexpensive and immediate quantification methods are needed to sort out sorghum grains for food, feed, and fuel.

Near-infrared (NIR) spectroscopy is a non-destructive, rapid, and low-cost method to estimate the biochemicals of cereal grains and differentiate them based on their chemical composition (Font et al., 2006; Hell et al., 2016; Caporaso et al., 2018). It is a feasible alternative to the time-inefficient and resource-intensive conventional methods of analysis, such as the Kjeldahl or high-performance liquid chromatography (HPLC) (Beć et al., 2021). A recent trend is to miniaturize NIR instruments that reduce the weight and cost (Wiedemair et al., 2019). However, Benchtop Fourier-Transform (FT) NIRS operates over the entire wavelength region with a high spectral resolution, and a good signal-to-noise ratio for yielding fast and accurate measurements (Beć et al., 2021). The NIR-based prediction models have been developed to estimate the chemical composition in grains of various crops (Pohl and Senn, 2011; Ferreira et al., 2015; Li et al., 2015; Kamboj et al., 2017). Previously reported NIR model calibrations using the whole grain spectra produce different results from using flour spectra and had moderate accuracy (De Alencar Figueiredo et al., 2006). Improved predictions are achievable by using the spectra of whole grains, when compared with the use of spectra collected from the flour samples (Beloshapka et al., 2016; Hu et al., 2021). It is also reported that the influence of grain hulls on the prediction of grain chemical content varies for different cultivars, and that milled grain flours might improve the prediction for grain chemical content (Wiedemair et al., 2019). However, due to the variation of hulls presence or absence in sorghum grains, the influence of hulls on the spectroscopic determination of cellulose, hemicellulose, lignin, and other components in sorghum grains is still unclear. The previous studies on how to effectively estimate chemical composition in different sorghum grain types are insufficient.

Thus, the objectives of this research were: (1) to evaluate the feasibility of using FT-NIR spectroscopy to determine a variety of chemical components, especially cellulose, hemicellulose, and lignin in sorghum grains; and (2) to evaluate the influence of four sample types (whole grains, flours, hulled grain flours, and hull-less grain flours) on the prediction of chemical components to improve the grain sorting efficiency for human food, animal feed, and biofuel.

## Materials and Methods

### Sampling and Measurements

A total of 20 hybrids of sorghum were grown at the experimental stations of China Agricultural University in Zhuozhou (Hebei province) and Jiexiu (Shanxi province), China, in 2017. In total 98 samples were collected from both the experimental stations (Supplementary Table 1). All the samples were first oven-dried at 45°C for 10 days to constant weight and then scanned as whole grains to acquire absorbance spectra using Thermo Antaris II FT-NIR spectrometer (Thermo Scientific Inc., Madison, WI, USA). Subsequently, the sorghum grain samples were milled to flours using a grinder, sifted through a 40–80 mesh screen, and scanned again as hulled or hull-less grain flours. The flours were stored at 4°C for chemical analysis. Starch, protein, fat, tannin, cellulose, hemicellulose, lignin, and ash contents were determined using these flour samples (Table 1).

**Table 1:** Descriptive statistics [minimum (min), maximum (max), mean, SD, and coefficient of variation (CV)] of laboratory analytical data comprising starch, protein, fat, tannin, cellulose, hemicellulose, lignin, and ash contents of distinct types of sorghum grains (whole grains, whole grain flours, hulled grain flours, and hull-less grain flours), used as reference data for calibration (C) and validation (V) subsets to construct PLSR models.

**Dataset**	**No. of samples**		**Statistics**	**Starch (g kg^−1^)**	**Protein (g kg^−1^)**	**Fat (g kg^−1^)**	**Tannin (g kg^−1^)**	**Cellulose (g kg^−1^)**	**Hemicellulose (g kg^−1^)**	**Lignin (g kg^−1^)**	**Ash (g kg^−1^)**
Whole grains	C	73	Min.	298.67	104.97	13.99	1.32	62.64	26.03	27.10	16.17
			Max.	784.79	185.01	58.99	19.92	216.86	90.43	87.68	36.37
			Mean ± SD	589.24 ± 98.25	136.21 ± 14.84	36.83 ± 8.95	12.99 ± 3.93	116.17 ± 30.27	56.04 ± 17.10	56.78 ± 16.17	23.44 ± 4.44
			CV (%)	16.67	10.89	24.32	30.27	26.06	30.52	28.48	18.94
	V	25	Min.	442.75	113.14	28.65	12.21	88.37	32.27	39.11	17.73
			Max.	762.90	160.67	49.05	17.06	134.26	87.89	77.39	32.33
			Mean ± SD	579.90 ± 75.10	136.50 ± 13.18	37.63 ± 5.67	14.95 ± 1.35	109.44 ± 13.86	64.55 ± 13.83	63.09 ± 12.06	25.17 ± 3.52
			CV (%)	12.95	9.66	15.07	9.08	12.66	21.42	19.12	14.00
Whole grain flours	C	73	Min.	298.67	104.97	13.99	1.32	62.64	26.03	27.10	16.17
			Max.	784.79	185.01	58.99	19.92	216.86	90.43	87.68	36.37
			Mean ± SD	589.24 ± 98.25	136.21 ± 14.84	36.83 ± 8.95	12.99 ± 3.93	116.17 ± 30.27	56.04 ± 17.10	56.78 ± 16.17	23.44 ± 4.44
			CV (%)	16.67	10.89	24.32	30.27	26.06	30.52	28.48	18.94
	V	25	Min.	442.75	113.14	28.65	12.21	88.37	32.27	39.11	17.73
			Max.	762.90	160.67	49.05	17.06	134.26	87.89	77.39	32.33
			Mean ± SD	579.90 ± 75.10	136.50 ± 13.18	37.63 ± 5.67	14.95 ± 1.35	109.44 ± 13.86	64.55 ± 13.83	63.09 ± 12.06	25.17 ± 3.52
			CV (%)	12.95	9.66	15.07	9.08	12.66	21.42	19.12	14.00
Hulled grain flours	C	45	Min.	376.98	104.97	13.99	11.01	75.84	26.03	31.39	22.11
			Max.	744.38	185.01	58.99	21.06	181.85	90.43	87.68	36.37
			Mean ± SD	546.95 ± 71.57	137.99 ± 17.13	36.41 ± 9.64	15.18 ± 2.33	113.30 ± 19.96	66.28 ± 13.10	67.90 ± 10.95	26.89 ± 3.51
			CV (%)	13.08	12.41	26.48	15.37	17.62	19.77	16.13	13.08
	V	16	Min.	433.30	120.96	26.55	12.56	88.37	51.72	57.68	22.17
			Max.	685.36	165.23	47.51	16.61	128.54	87.96	75.54	31.71
			Mean ± SD	563.41 ± 63.84	138.10 ± 12.86	35.38 ± 5.96	14.78 ± 1.09	109.02 ± 14.05	64.31 ± 9.38	66.80 ± 5.49	24.79 ± 2.22
			CV (%)	11.33	9.31	16.86	7.43	12.89	14.59	8.22	8.97
Hull-less grain flours	C	27	Min.	519.91	113.99	21.97	1.32	62.64	19.70	22.03	16.17
			Max.	784.79	156.99	53.18	19.75	216.86	49.37	57.03	25.83
			Mean ± SD	645.06 ± 77.75	134.08 ± 12.35	37.81 ± 7.55	11.10 ± 4.33	120.07 ± 38.30	35.30 ± 7.85	39.40 ± 7.44	20.12 ± 2.70
			CV (%)	12.05	9.21	19.97	39.03	31.89	22.25	18.90	13.44
	V	10	Min.	554.73	123.34	34.88	4.16	101.43	28.23	33.47	17.44
			Max.	735.26	146.47	49.05	15.62	174.50	46.44	41.43	23.11
			Mean ± SD	654.80 ± 64.49	133.47 ± 7.37	41.42 ± 5.15	12.34 ± 3.87	136.38 ± 25.16	35.44 ± 5.44	38.56 ± 2.64	19.75 ± 1.74
			CV (%)	9.85	5.52	12.44	31.36	18.45	15.35	6.86	8.82

### Determination of Chemical Components and Sample Classification

Starch content in sorghum grains was determined by the Anthrone colorimetric method and assayed using a UV-visible (UV-VIS) spectrometer (TU-1901, Beijing Purkinje Instruments Co., Ltd., Beijing, China) (Li et al., 2014). Crude protein was determined by Kjeldahl method through the measurement of total nitrogen. The content of crude fat was determined by ether extraction (Padmore, 1990). Tannin was extracted with dimethyl amide solution and tannic acid was used as a standard to determine the content of sorghum grain tannin (ISO, 1988). Cellulose, hemicellulose, and lignin were determined according to the modified National Renewable Energy Laboratory standards (NREL/TP-510-42618, Revised August 2012). Acid hydrolysis was done using the sulfuric acid (H_2_SO_4_). Acid insoluble lignin in the acid hydrolysis solution was determined to calculate the content of cellulose, hemicellulose, and lignin according to the following Equations (1–3), respectively.


(1) $$\begin{array}{r} \text{Cellulose }\left({\text{g kg}}^{-1}\right)=\text{Glucose }\left({\text{g kg}}^{-1}\right)\times 0.9\;\left(\text{correction rate}\right) \end{array}$$

(2) $$\begin{array}{r} \text{Hemicellulose }\left({\text{g kg}}^{-1}\right)=\left(\text{Xylose}\right)\;\left({\text{g kg}}^{-1}\right)+\text{Arabinose }\left({\text{g kg}}^{-1}\right)\times 0.88\;\left(\text{correction rate}\right) \end{array}$$

(3) $$\begin{array}{r} \text{Lignin}\left({\text{g kg}}^{-1}\right)=\text{Acid soluble lignin}\left({\text{g kg}}^{-1}\right)+\text{Acid insoluble lignin}\left({\text{g kg}}^{-1}\right) \end{array}$$

Additionally, we used perchloric acid as sorghum grains had a greater amount of starch and lignocellulosic components that were difficult to determine without using perchloric acid for acidification. After acidification, NREL standard methods were followed to determine cellulose, hemicellulose, and lignin content in sorghum grains (Sluiter et al., 2012). The ash content was determined by using a muffle furnace (VULCAN 3-550, Dentsply International Inc., York, PA, USA).

The samples were classified into three categories as food, feed, and fuel based on the available chemical content in the grains. The samples (*n* = 98) with equal or more than 15 g kg^−1^ (1.5%) tannin and 50 g kg^−1^ (5%) hemicellulose content were categorized as fuel samples (Waniska et al., 2016) (Table 1). The food and feed samples contained tannin and hemicellulose content < 15 and 50 g kg^−1^, respectively. The samples having starch < 650 g kg^−1^ (65%) were classified as feed and ≥ 650 g kg^−1^ (65%) were classified as food samples (Pontieri et al., 2010; Waniska et al., 2016; Beloshapka et al., 2016). Following the chemical properties, 14 samples were classified for food, 21 samples for feed, 38 samples for fuel, and 25 samples classified for both feed and fuel. The sample classification is shown in Supplementary Table 1.

### FT-NIR Spectroscopy and Data Processing

The FT-NIR spectra were scanned using the Thermo Antaris II FT-NIR spectrometer (Thermo Scientific Inc., Madison, WI, USA) prepared with an absorbance accessory. The grain and flour samples were put in a special Petri dish (black sides and transparent glass bottom) and fitted in the FT-NIR machine. Technical Quality (TQ) Analyst-pro software (Thermo Scientific Inc., Madison, WI, USA) was used to run the machine. Each spectrum had 64 scans at 4 cm^−1^ resolutions with the wavenumber range among 4,000–10,000 cm^−1^, including 1,557 spectral variables.

**Figure 1:**
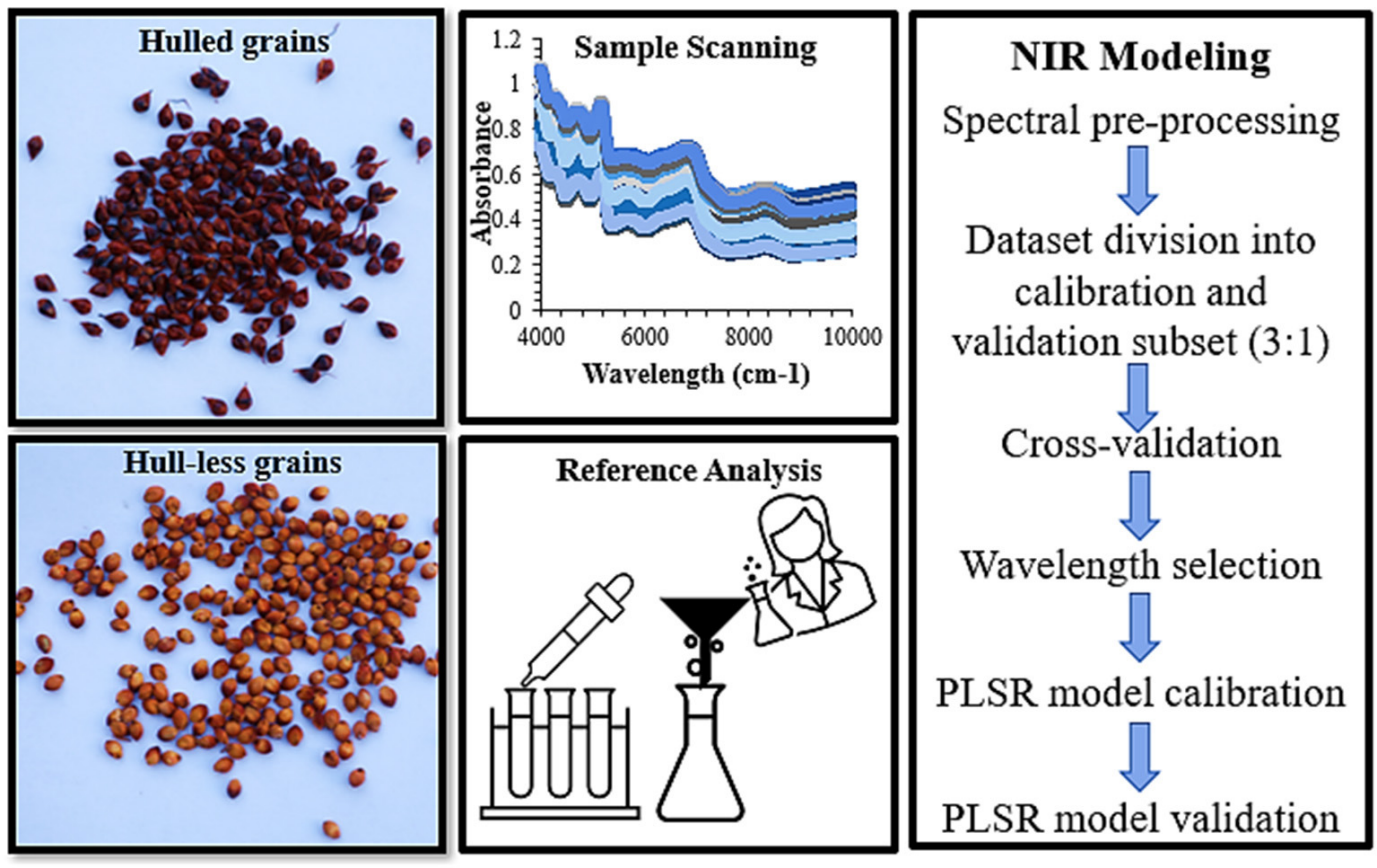
The morphological classification of sorghum grains by means of hulled and hull-less grains, also used as whole grains and flours by means of Fourier Transform near-infrared spectroscopy (FT-NIR) spectral data collection followed by reference analysis. The spectra were processed by combination of pre-treatments, cross-validated, calculation of principal components (PCs) by principal component analysis (PCA), divided into calibration and validation subsets and ultimately, partial least square regression (PLSR) models were built using FT-NIR spectral and reference data.

All obtained absorption spectra were analyzed using ChemDataSolution Version 3.1.0 (Dalian Chem. Data Solution Technology Co. Ltd., Dalian, China) (Figure 1). The raw spectra with the whole spectral range were preprocessed to remove random noise and small peaks by the combination of pretreatments: Multiplicative scatter correction (MSC) (Geladi and Macdougall, 1985) and standard normal variate (SNV) transformation (Brown et al., 2000) were used to remove the variations caused by instrument settings, sample, and environmental conditions. Norris derivative by means of Savitzky Golay (SG) method with 52 smoothing points (Brown and Wentzell, 2000) were used to resolve the spectra peak overlap and evacuate linear baseline drift with the selection of whole wavelength region (4,000–10,000 cm^−1^). The purpose of the pretreatments was to remove multiplicative and additive effects due to the machine settings and variations caused by the sample and environmental conditions. Based on different pretreatment combinations and performance, the models were cross-validated and the partial least square (PLS) factors were identified in the models for cross-validation (Geladi and Macdougall, 1985) (**Table 3**). To select the optimal number of PLS factors, the “leave-one-out” cross-validation method was used for developing PLS models to avoid over-fitting (Vehtari et al., 2017). Following the preprocessing treatments, the sample choice for the calibration and validation was carried out by the Kennard–Stone (KS) algorithm and Sample-set partitioning based on joint X–Y distances (SPXY) with the ratio of 3:1 (calibration:validation), respectively (Kennard and Stone, 1969) (Table 2). While KS algorithm only concerns the similarity between independent variables of the two subsets, SPXY combines independent and dependent variables (Galvão et al., 2005). All the spectral data were mean-centered before calibration and the competitive adaptive reweighted sampling (CARS) method was used to select the wavelength (Li et al., 2009; Wang et al., 2015). The regression coefficients of PLS models were used as an index for evaluating the importance of each wavelength (Li et al., 2009). The PLS regression was used to construct the calibration models for the biochemical evaluation of grains (Figure 1).

**Table 2:** The PLSR followed by mathematical treatments of spectra to remove random noise and to construct FT-NIR models for biochemical components (starch, protein, fat, tannin, cellulose, hemicellulose, lignin, and ash) of whole grains, whole grain flours, hulled, and hull-less grain flours of sorghum.

**Dataset**	**Biochemical component**	**Mathematical treatments of spectra** **^a^**				
		**Pretreatment**	**Smoothing**	**Derivative**	**Variable selection**	**Sample selection**
Whole grains	Starch	MSC	SG52	Second	CARS	SPXY
	Protein	MSC	SG52	Second	CARS	SPXY
	Fat	SNV	SG52	Second	CARS	SPXY
	Tannin	SNV	SG52	Second	CARS	SPXY
	Cellulose	SNV	SG52	Second	CARS	SPXY
	Hemicellulose	MSC	SG52	Second	CARS	SPXY
	Lignin	SNV	SG52	First	CARS	KS
	Ash	MSC	SG52	Second	CARS	SPXY
Whole grain flours	Starch	SNV	SG52	Second	CARS	KS
	Protein	MSC	SG52	Second	CARS	SPXY
	Fat	MSC	SG52	Second	CARS	KS
	Tannin	SNV	SG52	Second	CARS	KS
	Cellulose	SNV	SG52	Second	CARS	SPXY
	Hemicellulose	SNV	SG52	Second	CARS	SPXY
	Lignin	MSC	SG52	Second	CARS	SPXY
	Ash	MSC	SG52	Second	CARS	SPXY
Hulled grain flours	Starch	MSC	SG52	Second	CARS	SPXY
	Protein	SNV	SG52	Second	CARS	SPXY
	Fat	SNV	SG52	Second	CARS	KS
	Tannin	MSC	SG52	Second	CARS	SPXY
	Cellulose	MSC	SG52	Second	CARS	KS
	Hemicellulose	SNV	SG52	Second	CARS	SPXY
	Lignin	SNV	SG52	Second	CARS	SPXY
	Ash	MSC	SG52	Second	CARS	SPXY
Hull-less grain flours	Starch	MSC	SG52	First	CARS	SPXY
	Protein	SNV	SG52	First	CARS	SPXY
	Fat	SNV	SG52	Second	CARS	SPXY
	Tannin	SNV	SG52	First	CARS	SPXY
	Cellulose	MSC	SG52	Second	CARS	KS
	Hemicellulose	SNV	SG52	Second	CARS	SPXY
	Lignin	MSC	SG52	Second	CARS	SPXY
	Ash	MSC	SG52	First	CARS	SPXY

a *MSC, Multiplicative scatter correction; SNV, Standard normal variate; SG, Savitzky Golay; CARS, Competitive adaptive reweight sampling method; SPXY, Sample set partitioning based on joint x–y distances; KS, Kennard–stone algorithm; methods to separate samples for calibration and validation*.

First, all the values of each biochemical were built-in the model to gain an understanding of the initial standard error and their correlation coefficients. Outliers were detected from the first calibrated model by calculating the CI (95%) of the first two principal components (PCs) from principal component analysis (PCA) plots in Moli Software (Dalian Chem. Data Solution Technology Co. Ltd., Dalian, China) and the samples out of this interval were removed individually from the sample set of each relevant model. The models were recalibrated by following the above spectra preprocessing methods to lower the number of PCs.

### Performance Index of Partial Least Squares Regression Models

The performance of multivariate calibrations was evaluated according to the determination coefficient (*R*^2^), root mean square error of calibration (RMSEc), root mean square error of validation (RMSEv), and the ratio of prediction to deviation (RPD) for the calibration and validation subsets. These parameters were used to determine the potential of the partial least squares regression (PLSR) models for the determination of biochemical components in sorghum grains (Wu et al., 2015; Yang et al., 2016) (Table 3). In model development and applications, *R*^2^ > 0.95 and RPD > 4 indicates that the model could predict efficiently, 0.95 > *R*^2^ > 0.9 and 4 > RPD > 3 regarded as successful calibration, 0.9 > *R*^2^ > 0.8 and 3 > RPD > 2.25 considered satisfactory, and 0.8 > *R*^2^ > 0.7 and 2.25 > RPD > 1.75 is regarded good for preliminary screenings (Malley et al., 2004; Arana et al., 2005) (Table 3).

**Table 3:** Statistics of PLSR models constructed using FT-NIR spectroscopy to determine starch, protein, fat, tannin, cellulose, hemicellulose, lignin, and ash in distinct types of sorghum grains (whole grains, whole grain flours, hulled, and hull-less grain flours) used for calibration, validation, and cross-validation subsets.

**Dataset**	**Biochemical component**	**PC**	**Calibration**			**Cross-validation**			**Validation**			
			***R*** ^**2**^	**RMSEc**	**SD**	***R*** ^**2**^	**RMSEcv**	**RPD**	***R*** ^**2**^	**RMSEv**	**RPD**	**SD**
Whole grains	Starch	8	0.98	10.90	98.25	0.92	26.29	3.73	0.88	24.94	3.01	75.10
	Protein	8	0.97	2.12	14.84	0.90	4.65	3.18	0.89	4.23	3.11	13.18
	Fat	8	0.98	1.06	8.95	0.91	2.56	3.48	0.91	1.76	3.21	5.67
	Tannin	7	0.98	0.04	0.39	0.93	0.09	3.96	0.72	0.06	1.94	0.13
	Cellulose	8	0.99	1.45	27.03	0.96	4.89	5.52	0.88	4.68	2.96	13.86
	Hemicellulose	9	0.99	0.47	17.10	0.98	1.96	8.69	0.97	2.23	6.18	13.83
	Lignin	7	0.97	2.55	16.17	0.94	3.75	4.31	0.93	3.07	3.91	12.06
	Ash	6	0.98	0.54	4.44	0.94	1.00	4.43	0.93	0.88	3.96	3.52
Whole grain flours	Starch	6	0.96	16.83	96.32	0.91	28.60	3.36	0.90	23.01	3.05	70.37
	Protein	5	0.98	2.05	15.09	0.96	2.88	5.23	0.96	2.37	5.35	12.73
	Fat	9	0.97	1.22	8.00	0.84	3.14	2.54	0.90	2.68	3.17	8.53
	Tannin	7	0.96	0.07	0.38	0.91	0.86	2.73	0.91	0.09	3.27	0.30
	Cellulose	7	0.99	2.04	25.83	0.94	6.14	4.20	0.92	5.91	3.58	21.20
	Hemicellulose	8	0.99	1.29	16.88	0.97	2.70	6.23	0.99	2.44	6.89	16.84
	Lignin	8	0.99	0.76	16.08	0.98	1.91	8.39	0.97	1.97	6.92	13.65
	Ash	5	0.98	2.05	15.09	0.96	2.88	5.23	0.96	2.37	5.35	12.73
Hulled grain flours	Starch	6	0.99	6.75	71.57	0.96	13.29	5.38	0.92	18.61	3.42	63.84
	Protein	8	0.99	1.09	17.13	0.98	2.14	7.97	0.98	1.79	7.17	12.86
	Fat	6	0.98	0.10	0.96	0.92	0.27	3.48	0.93	0.16	3.62	0.59
	Tannin	8	0.99	0.01	0.23	0.94	0.05	4.32	0.90	0.04	2.74	0.10
	Cellulose	9	0.99	1.54	19.96	0.93	4.91	4.05	0.90	4.25	3.30	14.05
	Hemicellulose	9	0.99	0.73	11.71	0.96	2.24	5.21	0.93	2.75	3.40	9.38
	Lignin	7	0.99	0.87	9.54	0.94	2.22	4.28	0.95	1.40	3.91	5.49
	Ash	7	0.99	0.31	3.51	0.96	0.66	5.33	0.97	0.40	5.56	2.22
Hull-less grain flours	Starch	5	0.97	1.24	7.90	0.88	2.62	3.01	0.93	1.57	3.92	6.19
	Protein	6	0.99	1.11	12.35	0.97	2.06	5.98	0.94	1.85	3.97	7.37
	Fat	9	0.99	0.01	0.75	0.97	0.11	6.48	0.97	0.08	6.28	0.51
	Tannin	8	0.97	0.06	0.44	0.90	0.13	3.36	0.99	0.04	8.46	0.38
	Cellulose	9	0.99	1.04	38.30	0.97	5.79	6.61	0.97	4.11	6.11	25.16
	Hemicellulose	9	0.99	0.17	7.85	0.98	1.08	7.26	0.97	0.92	5.86	5.44
	Lignin	7	0.99	0.31	7.44	0.98	1.08	6.87	0.97	0.47	5.55	2.64
	Ash	6	0.98	0.32	2.70	0.93	0.68	3.94	0.88	0.60	2.88	1.74

*PC, number of principal components; R^2^, the co-efficient of determination; RMSEc, root mean square error of calibration; RMSEv, root mean square error of validation; RMSEcv, root mean square error of cross-validation; SD, standard deviation; RPD, ratio of prediction to deviation*.

## Results

### Chemical Properties and Grain Grading

The minimum contents of tannin and high starch in food grains were 11.66 and 695.57 g kg^−1^, respectively found in 622A × J7645Z hybrid (Table 1; Supplementary Table 1). The minimum hemicellulose content in the food samples was 29.92 g kg^−1^ in 622A × J7645Z (Table 1; Supplementary Table 1). The Awanlek hybrid contains minimum tannin and starch content of 6.44 and 597.11 g kg^−1^, respectively, useful for feed (Table 1; Supplementary Table 1). Fuel samples contain maximum cellulose and hemicellulose content (125.86 and 80.35 g kg^−1^, respectively) (X098 and NW3) (Table 1; Supplementary Table 1). J7645Z hybrid contains maximum starch content that is useful for fuel samples (730.68 g kg^−1^) (Table 1; Supplementary Table 1). The maximum content of protein, fat, lignin, and ash in fuel samples were 169.44, 44.91, 75.99, and 30.59 g kg^−1^, respectively. The samples used for both feed and fuel, contain low tannin and starch (useful for feed) but high cellulose, hemicellulose, and lignin content (useful for fuel). The minimum starch and tannin content in combined feed/fuel samples were 498.65 and 13.85 g kg^−1^, respectively (AMP450 × NW3) (Table 1; Supplementary Table 1). Moreover, the average chemical properties for different morphological types of sorghum grains revealed that hull-less grain flours had high levels of starch content (647.4 g kg^−1^) compared with the whole grains/flours (585.6 g kg^−1^) and hulled grain flours (548.1 g kg^−1^) (Table 1; Supplementary Table 1). By contrast, the content of protein and fat were not significantly different for all types of datasets. Tannin content was found higher in hulled flours (15.0 g kg^−1^) and lower in hull-less flours (11.1 g kg^−1^) compared to whole grains/flours samples (13.6 g kg^−1^) (Table 1). The average cellulose content was the highest in hull-less flours (124.3 g kg^−1^) and the least in hulled flours (116.6 g kg^−1^) while on the contrary, average hemicellulose content was the highest in hulled flours (66.4 g kg^−1^) and the least in hull-less flours (35.3 g kg^−1^) compared with whole grains/flours. Similarly, the average lignin and ash content were also high in hulled flours (68.2 and 26.3 g kg^−1^, respectively) (Table 1).

The coefficient of variation (CV) for the biochemical components in different sorghum morphological types (grains, flours, hulled flours, and hull-less flours) used as reference data for calibration and validation subsets are shown in Table 1. Biochemical data used in the whole grains and flours model showed that hemicellulose and lignin have the highest CVs for both the calibrations (30.52 and 28.48%, respectively) and validation subsets (21.42 and 19.12%, respectively). Tannin has CV for both calibration and validation (30.27 and 9.08%, respectively), followed by cellulose (26.06 and 12.66%, respectively), fat (24.32 and 15.07%, respectively), ash (18.94 and 14.0%, respectively), starch (16.67 and 12.95%, respectively), and the smallest by protein (10.89 and 9.66%, respectively). The biochemical data used in the hulled and hull-less grain flour models showed similar ranges of CV (Table 1).

### Spectral Pretreatment and Models Performance

**Figure 2:**
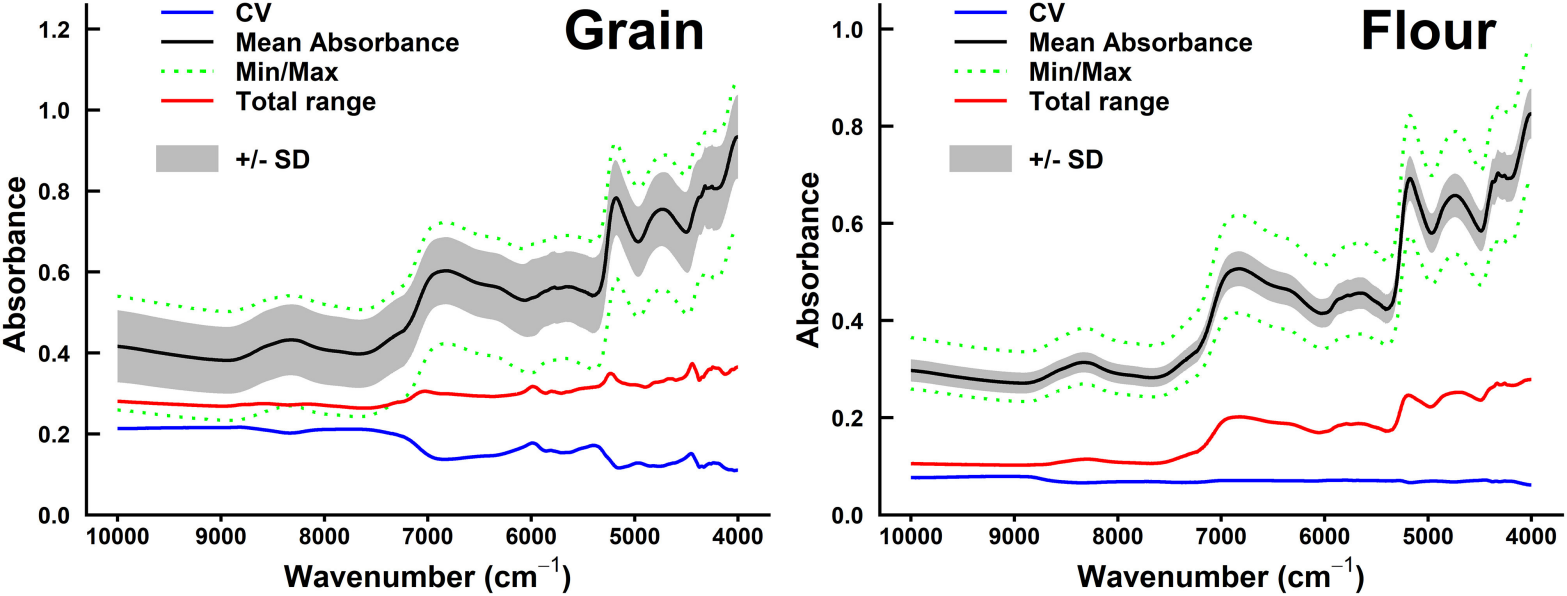
Summary of spectral absorbance and characteristic wavelengths distribution measurements for sorghum whole grains vs. flours sample set (*n* = 98). The range of coefficient variation (CV, blue line), mean absorbance (black line), minimum/maximum (green dotted line) spectral absorbance, total range of absorbance measurements (red line) for each wavelength (cm^−1^), and SD+/– SD between sample sets were calculated.

**Figure 3:**
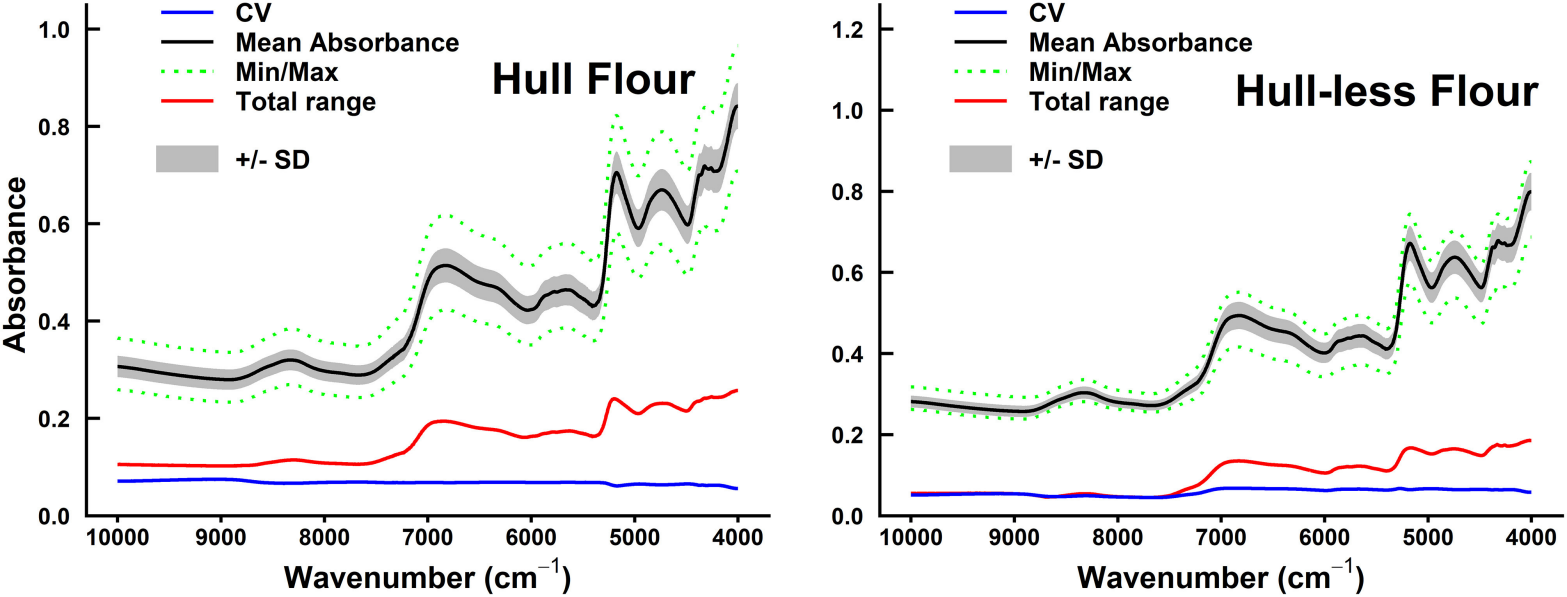
Summary of spectral absorbance and characteristic wavelengths distribution measurements for sorghum hulled (*n* = 61) vs. hull-less grain flours sample set (*n* = 37). The CV, coefficient of variation (blue line) (SD/mean), mean absorbance (black line), minimum/maximum (green dotted line) spectral absorbance, total range of absorbance measurements (red line) for each wavelength (cm^−1^), and +/– SD between sample sets were calculated.

The mean, minimum, and maximum absorbance of sorghum grain samples are shown in Figures 2, 3. The minimum absorption was found between 10,000 and 9,000 cm^−1^ wavelength regions. The maximum absorption peaks were found in the 9,000–4,000 cm^−1^ wavelength regions and showed a common phenomenon in whole grains, whole grain flours, hulled, and hull-less flours. The absorbance of whole-grain flours was the least among all types of samples. Whole grain flours absorbed at around 10,000 cm^−1^, with values ranging from 0.25 to 0.4 (Figure 2) that was adjacent to the absorption of hull-less and hulled grain flour which were absorbed between 0.4 and 0.43, respectively (Figure 3).

The development of PLS regression models for each biochemical component of sorghum grains were performed to minimize the effect of the change in the baseline drift. Most of the models performed best in a combination of second derivative SG52, CARS, and SPXY methods, except for lignin in whole grains and starch, protein, tannin, and ash in hull-less grain flours that performed best with first derivative and KS sample selection method. All the models were constructed by critically evaluating different spectral pretreatments that gave the higher R^2^c (≥0.96), R^2^v (≥0.72), R^2^cv (≥0.84), RPDv (≥1.9), and RPDcv (≥2.5) (Table 3). A *T*-test was applied to determine the prediction accuracy of the models for different sample formats (Supplementary Figures 1, 2).

### Comparison of the Models of Whole Grains and Flours

**Figure 4:**
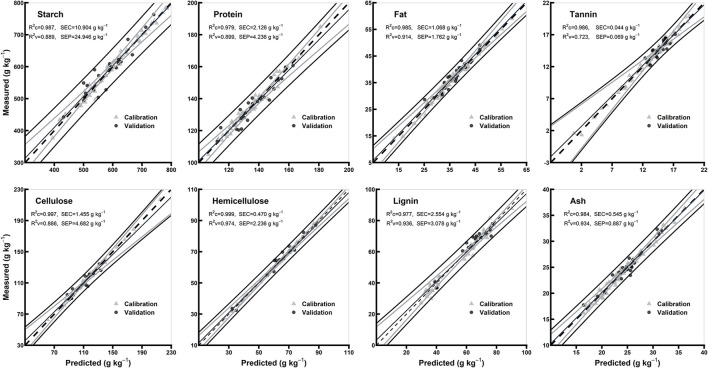
Comparison of scatter plots of measured vs. predicted values (g kg^−1^) of the whole grains for starch, protein, fat, tannin, cellulose, hemicellulose, lignin, and ash of sorghum grains for the external validation subsets based on PLSR models. The black and gray lines in plot showed mean, maximum, and minimum values while dotted lines showed average chemical composition between calibration and validation sample set.

**Figure 5:**
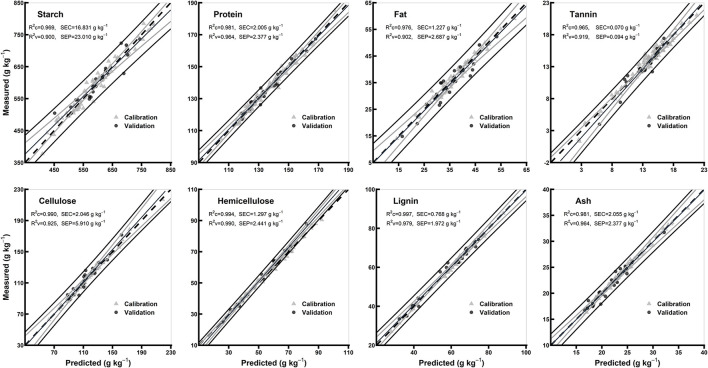
Comparison of scatter plots of measured vs. predicted values (g kg^−1^) of the whole grain flours for starch, protein, fat, tannin, cellulose, hemicellulose, lignin, and ash of sorghum grains for the external validation subsets based on PLSR models. The black and gray lines in plot showed mean, maximum, and minimum values while dotted lines showed average chemical composition between calibration and validation sample set.

Table 3 shows model statistics for all biochemical components in the models of whole grains and flours. In the whole grain models, the R^2^c for all the chemical components were ≥0.97, R^2^v were ≥0.72 and R^2^cv were ≥0.90, while RPDc were ≥1.94 and RPDcv were ≥3.18. Based on the highest R^2^c (0.99) and RPDc (6.18), hemicellulose model was considered best among the other chemical components in whole grain models (Table 3). In flours models, the R^2^c for all the chemical components were ≥0.96, R^2^v were ≥0.90, and R^2^cv were ≥0.84, while RPDc were ≥3.05 and RPDcv were ≥2.54. Hemicellulose and lignin were considered best among the other chemical components in flours models based on the highest R^2^c (0.99 for both) and RPDc (6.89 and 6.92, respectively) (Table 3). Overall, the model validation for flours was better than the whole grains. Figures 4, 5 show the scatter plots for predicted and measured values of different biochemical components in the whole grains and flours, respectively.

### Comparison of the Models of Hulled and Hull-Less Flours

**Figure 6:**
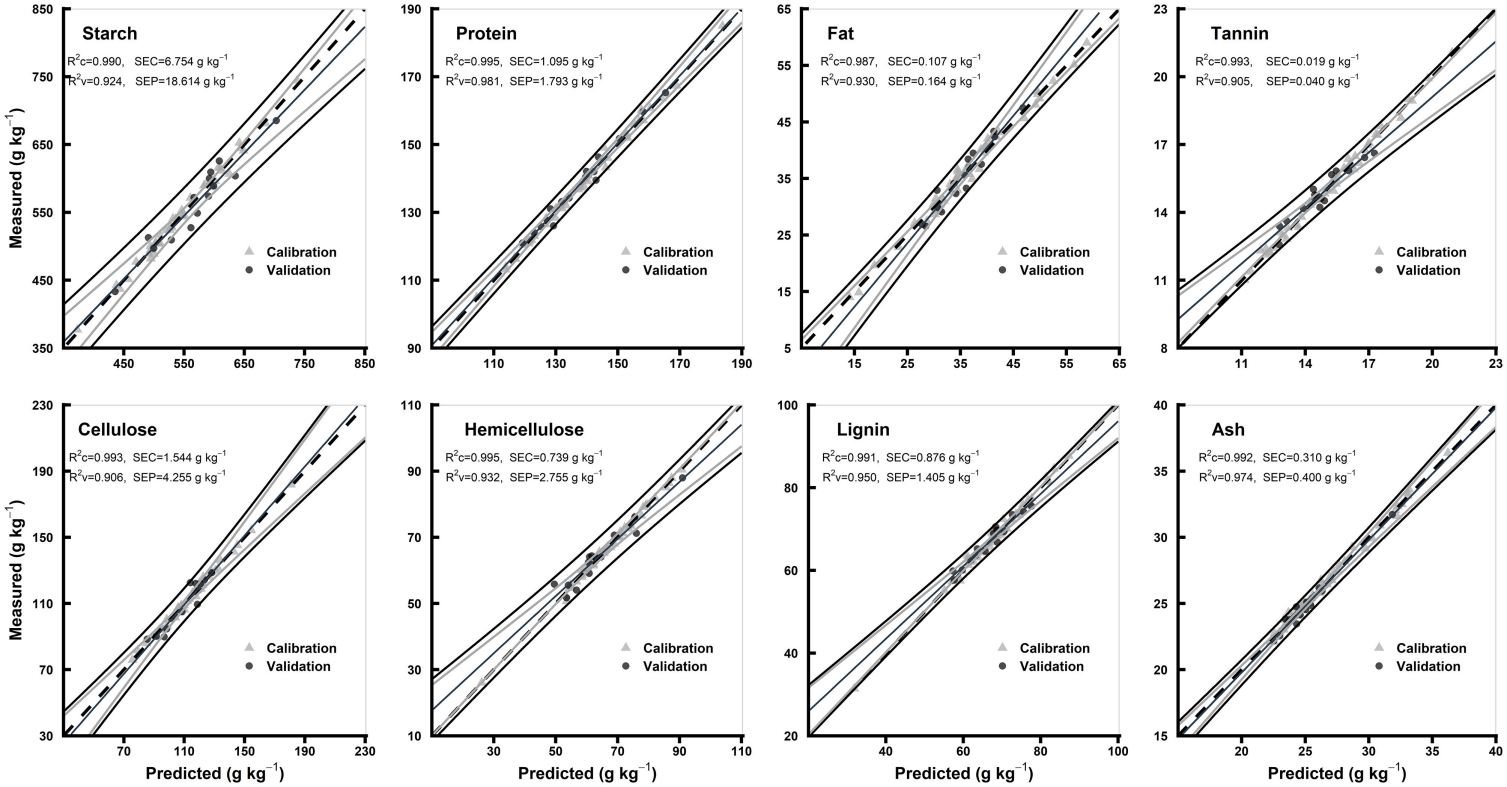
Comparison of scatter plots of measured vs. predicted values (g kg^−1^) of the hulled grain flours for starch, protein, fat, tannin, cellulose, hemicellulose, lignin, and ash of sorghum grains for the external validation subsets based on PLSR models. The black and gray lines in plot showed mean, maximum, and minimum values while dotted lines showed average chemical composition between calibration and validation sample set.

**Figure 7:**
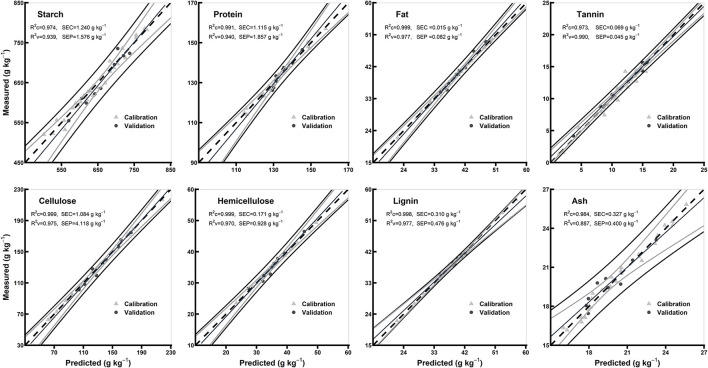
Comparison of scatter plots of measured vs. predicted values (g kg^−1^) of the hull-less grain flours for starch, protein, fat, tannin, cellulose, hemicellulose, lignin, and ash of sorghum grains for the external validation subsets based on PLSR models. The black and gray lines in plot showed mean, maximum, and minimum values while dotted lines showed average chemical composition between calibration and validation sample set.

The statistics for all the biochemical components in the models of hulled and hull-less grain flours are shown in Table 3. In hulled grain flours models, the R^2^c for all the chemical components were ≥0.98, R^2^v were ≥0.90, and R^2^cv were ≥0.92, while RPDc were ≥2.74 and RPDcv were ≥3.48. Based on the highest R^2^c (0.99) and RPDc (7.17), protein model was considered the best among other chemical components in hulled grain flours models (Table 3). In hull-less grain flours models, the R^2^c for all the chemical components were ≥0.97, R^2^v were ≥0.93 and R^2^cv were ≥0.88, while RPDc were ≥2.88 and RPDcv were ≥ 3.01. Fat, tannin, and cellulose were considered the best among other chemical components in flours models based on the highest R^2^c (0.99, 0.97, and 0.99, respectively) and RPDc (6.28, 8.46, and 6.1, respectively) (Table 3). Figures 6, 7 show the scatter plots for predicted and measured values of different biochemical components in the hulled and hull-less and flours, respectively. The hulled grain flours allowed for the best prediction for protein, whereas the hull-less grain flours allowed for the best prediction for tannin, cellulose, and hemicellulose (Figures 6, 7).

## Discussion

### Chemical Properties and Food, Feed, and Fuel Grading

In this study, sorghum grains were graded based on their respective chemical content for food, feed, and fuel. Out of the 20 hybrids of sorghum grains used in this study, hull-less hybrids which include 622A × Awanlek, 622A × J7645Z, and 624A × J7645Z contained high starch and cellulose while low tannin and hemicellulose content and were considered suitable for food. The protein content was high in the food samples compared with the feed samples. Hull-less hybrids (Awanlek, 624A × Awanlek, AMP450 × Awanlek, and AMP450 × J7645Z) having low tannin, starch, cellulose, and hemicellulose content were used for feed. Similar to previous studies, the content of fat, lignin, and ash in both food and feed samples (hull-less) was nearly in the same range (Pontieri et al., 2010; Waniska et al., 2016; Beloshapka et al., 2016). Due to the non-digestible nature of hemicellulose and the bitter taste of tannin, these components are not desirable for food and feed, which leads to conclude that hull-less samples can be used as food (Table 1). The overall content of lignocelluloses in feed was higher than the food but significantly lower than the fuel grain samples. Based on required chemical components, whole grains/flours can be used as animal feed.

Moreover, owing to the high content of tannin, lignocelluloses, and ash, hulled grain hybrids (NW2, NW3, X098, 624A × NW2, AMP450 × NW2, 624A × X098, and 622A × NW3) were used as fuel. However, 622A × NW2, 622A × X098, AMP450 × X098, 624A × NW3, and AMP450 × NW3 hybrids were used for both feed and fuel due to desirable chemical content for feed and fuel (low tannin and high hemicellulose). The insoluble dietary fibers especially hemicelluloses are not seen as desirable as food (Fuller et al., 2016; Qiu et al., 2017; Stamenković et al., 2020). According to the previous studies, the hulled grains with an increasing tannin, cellulose, hemicellulose, lignin, and ash were used as an ideal additive for biofuel production (Table 1) (Eggum et al., 1983; Corredor et al., 2005; Stamenković et al., 2020). In this study, insoluble dietary fibers were found higher in the hulled grains with increasing content of starch, tannin, and ash and proposed as a supreme stabilizer for biofuel. Similar to the previous studies, hulled grains contained less starch while high fibers content (hemicellulose and lignin), tannin and ash compared with hull-less grains (Eggum et al., 1983).

A large CV in the sample datasets for starch, protein, fat, tannin, cellulose, hemicellulose, lignin, and ash was observed in this study. The previous studies found that the CV of starch, protein, fat, tannin, cellulose, hemicellulose, lignin, and ash in sorghum grains for bulk number of samples were 5.32, 13.2, 16.3, 9.09, 32, 30, 19, and 9.41%, respectively (De Alencar Figueiredo et al., 2006; Hill et al., 2012; Li et al., 2014; Wang et al., 2020). Compared with the previous studies, the higher CVs in this study indicate a wide variation among the sorghum grain samples and a large potential of model robustness when more samples from more hybrids and from more regions to be included.

### PLSR Model Performance

For each chemical component and morphological type (whole grains, whole grain flours, hulled grain flours, and hull-less grain flours) of sorghum grain samples, spectral preprocessing yielded different results in the PLSR models. The predicted and reference values of whole grains, whole grain flours, hulled grain flours, and hull-less grain flours were consistent (Figures 4–7, respectively). Several physical phenomena contribute to the additive and multiplicative scatter effects, caused by the differences in the grain surface structure, i.e., hulled or hull-less grains. The spectral data were preprocessed to eliminate the influence of light scattering from the non-homogeneity of the sample particle distribution (Pohl and Senn, 2011). MSC can be considered as a suitable method when working with the samples constituted by particle size because it varies according to the grain hardness and uniformity to random noise (Sampaio et al., 2018). MSC has a tendency to produce outliers, while SNV induce curved structures derived from the treated spectra (Fearn et al., 2009). Both MSC and SNV methods assisted in getting models performance with different combinations of treatments for different chemicals.

The variations within and across the calibration and validation processes were maximized and repeated several times using the SPXY and KS sample selection methods (Vehtari et al., 2017). To demonstrate the effect of SPXY and KS optimization on the partitioning of calibration and validation subsets, PCA was applied and results indicate that the SPXY and KS methods could efficiently optimize NIRS results as well as the analytical properties of both the calibration and validation subsets (Table 3). These distributions and optimizations led to the good performance of FT-NIR models.

Lignin in whole grain flours showed reasonably accurate results using the first derivative and KS sample selection method while all other chemical components in whole grains showed precise results with a combination of the second derivative and SPXY sample selection method. Likewise, starch, protein, tannin, and ash in hull-less flours showed good results using the first derivative while all other morphological types of grains showed accurate results using the second derivative. Starch, fat, and tannin in whole grain flours, fat, and cellulose in hulled grain flours, and cellulose in hull-less grain flours showed reasonably precise results using KS sample selection method, compared with other chemicals in all the morphological types of sorghum grains (Table 2). Similar to the previous studies, spectral pretreatment especially derivative and spectra smoothing improved the models' performance and quality (Wolfrum et al., 2013).

The values of *R*^2^ and RPD for all the models were high enough to use them for future predictions (Malley et al., 2004). In contrary to the previous studies, the prediction accuracy (paired *t*-test between the measured and predicted values) of both the models of whole grains and flours was not different (Flinn et al., 1998; Garnsworthy et al., 2000; De Alencar Figueiredo et al., 2006) (Table 3; Figures 4, 5). However, it is suggested that the whole grains can be used instead of grinding because the goodness of fit of both grains and flours models showed. Moreover, the stiff texture of the hulls has a significant effect on the performance of calibration of biochemical components, indicating that the hulls may distort spectral information (De Alencar Figueiredo et al., 2006; Guindo et al., 2016) (Table 3; Figures 6, 7). To determine the prediction accuracy of the models, *t*-test was applied between the morphological types of sorghum grains, i.e., grains vs. flours, hulls vs. hull-less. It showed that the flour models were significantly better for prediction of protein, tannin, and ash while the hull-less models were significantly better for prediction of fat, hemicellulose, and lignin (Supplementary Figures 1, 2). However, the performance index of all the models demonstrated that these models performed well in the sample set and could be used for the estimation of future screenings and predictions, efficiently (Table 3).

The previous research showed the potential for analysis for the major chemical components and forms of grains according to their needs and interest. Nevertheless, models determining the content of the chemical components especially cellulose, hemicellulose, and lignin in sorghum grain flours are rarely investigated, nor systematically compared for different grain types. FT-NIR spectroscopy for grading grains based on grain quality parameters is preferable because it is non-destructive and non-hazardous. RPD and RPDcv showed that all the models could successfully predict the biochemicals, suggesting that the FT-NIR spectroscopy is a promising tool for predictions of sorghum grain biochemicals used for food, feed, and fuel (Table 3). The models presented in this paper can be applied to the industry level for producers to select grains with outstanding potential for food, feed, and fuel productions. This is a high throughput method with high accuracy as compared with the wet chemical determination methods to determine the desired chemical components for human food, animal feed, and biofuel. To improve model performance, it is necessary to extend the sample size over years so that prediction could be improved because the environmental factors induce high influence on the properties and chemical components of sorghum grains.

## Conclusion

Biochemical components of sorghum were successfully predicted for enhancing grain sorting efficiency for food, feed, and fuel using FT-NIR spectroscopy. The PLS regression models allowed to screen and predict chemical components especially lignocellulose (cellulose, hemicellulose, and lignin) predictions that were rarely reported. Cellulose, and hemicellulose and lignin concentrations could be predicted using NIR data measured on whole grains, with high accuracy (*R*^2^ > 0.88); thus, whole grains are recommended instead of flours to save grinding time and labor. NIR-based PLS models for varied compositions of hulled and hull-less grain flours compared to the wet chemical procedures are desirable. The established PLSR models could enable food, feed, and fuel producers to efficiently evaluate a large number of samples by predicting the required biochemical components in sorghum grains.

## Data Availability Statement

The original contributions presented in the study are included in the article/Supplementary Material, further inquiries can be directed to the corresponding author/s.

## Author Contributions

IE: conceptualization, methodology, and writing—original draft. SH: formal analysis and investigation. WL: data analysis, visualization, and results interpretation. NH: results interpretation and writing. CT, SL, and BD: resources. ML: software. GX: data curation and funding acquisition. KY: supervision, data analysis, results interpretation, writing, and funding acquisition. All authors contributed to the article and approved the submitted version.

## Funding

This research was supported by the Department of Energy Conservation and Technology Equipment of China's National Energy Administration [Science and Technology Department, No. (2012) 32] and funded by China Datang New Energy Co. LTD, Henan Tianguan Group Co., Ltd., and the CAU Basic Scientific Research Fund (2020RC037).

## Conflict of Interest

The authors declare that the research was conducted in the absence of any commercial or financial relationships that could be construed as a potential conflict of interest.

## Publisher's Note

All claims expressed in this article are solely those of the authors and do not necessarily represent those of their affiliated organizations, or those of the publisher, the editors and the reviewers. Any product that may be evaluated in this article, or claim that may be made by its manufacturer, is not guaranteed or endorsed by the publisher.
