# Supplementary material for: Sorghum Grains Grading for Food, Feed, and Fuel Using NIR Spectroscopy
Source: Front Plant Sci. 2021 Sep 16;12:720022. doi: 10.3389/fpls.2021.720022 (PMC8481643; doi:10.3389/fpls.2021.720022)

**Supplementary**

Table S1. List of sorghum grain samples used in this study and the classification of their usages, collected from field experiment at Zhuozhou (Hebei province) and Jiexiu (Shanxi province), China, in 2017.

| **Sample No.** | **Hybrid No.** | **ID** | **Experimental area** | **^a^CMS** | **^b^CMF** | **Character** | **^c^Category** |
| --- | --- | --- | --- | --- | --- | --- | --- |
| 1 | 1 | A248 | Zhuozhou |  | Awanlek | Hull-less | Feed |
| 2 |  | B087 | Zhuozhou |  | Awanlek | Hull-less | Feed |
| 3 |  | C019 | Zhuozhou |  | Awanlek | Hull-less | Feed |
| 4 |  | A248 | Jiexiu |  | Awanlek | Hull-less | Feed |
| 5 |  | B087 | Jiexiu |  | Awanlek | Hull-less | Feed |
| 6 |  | C019 | Jiexiu |  | Awanlek | Hull-less | Feed |
| 7 | 2 | A210 | Zhuozhou |  | NW2 | Hull | Fuel |
| 8 |  | C152 | Zhuozhou |  | NW2 | Hull | Fuel |
| 9 |  | B119 | Jiexiu |  | NW2 | Hull | Fuel |
| 10 |  | C152 | Jiexiu |  | NW2 | Hull | Fuel |
| 11 | 3 | A009 | Zhuozhou |  | X098 | Hull | Fuel |
| 12 |  | B229 | Zhuozhou |  | X098 | Hull | Fuel |
| 13 |  | C115 | Zhuozhou |  | X098 | Hull | Fuel |
| 14 |  | A009 | Jiexiu |  | X098 | Hull | Fuel |
| 15 |  | B229 | Jiexiu |  | X098 | Hull | Fuel |
| 16 |  | C115 | Jiexiu |  | X098 | Hull | Fuel |
| 17 | 4 | C117 | Zhuozhou |  | NW3 | Hull | Fuel |
| 18 |  | B247 | Jiexiu |  | NW3 | Hull | Fuel |
| 19 |  | C117 | Jiexiu |  | NW3 | Hull | Fuel |
| 20 | 5 | B188 | Zhuozhou | 622A | Awanlek | Hull-less | Food |
| 21 |  | C133 | Zhuozhou | 622A | Awanlek | Hull-less | Food |
| 22 |  | A155 | Jiexiu | 622A | Awanlek | Hull-less | Food |
| 23 |  | B188 | Jiexiu | 622A | Awanlek | Hull-less | Food |
| 24 |  | C133 | Jiexiu | 622A | Awanlek | Hull-less | Food |
| 25 | 6 | A288 | Zhuozhou | 624A | Awanlek | Hull-less | Feed |
| 26 |  | B089 | Zhuozhou | 624A | Awanlek | Hull-less | Feed |
| 27 |  | C308 | Zhuozhou | 624A | Awanlek | Hull-less | Feed |
| 28 |  | A288 | Jiexiu | 624A | Awanlek | Hull-less | Feed |
| 29 |  | B089 | Jiexiu | 624A | Awanlek | Hull-less | Feed |
| 30 |  | C308 | Jiexiu | 624A | Awanlek | Hull-less | Feed |
| 31 | 7 | B139 | Zhuozhou | AMP450 | Awanlek | Hull-less | Feed |
| 32 |  | C335 | Zhuozhou | AMP450 | Awanlek | Hull-less | Feed |
| 33 |  | A211 | Jiexiu | AMP450 | Awanlek | Hull-less | Feed |
| 34 |  | B139 | Jiexiu | AMP450 | Awanlek | Hull-less | Feed |
| 35 | 8 | B255 | Zhuozhou | 622A | NW2 | Hull | Fuel/feed |
| 36 |  | A073 | Jiexiu | 622A | NW2 | Hull | Fuel/feed |
| 37 |  | B255 | Jiexiu | 622A | NW2 | Hull | Fuel/feed |
| 38 |  | C160 | Jiexiu | 622A | NW2 | Hull | Fuel/feed |
| 39 | 9 | A279 | Zhuozhou | AMP450 | NW2 | Hull | Fuel |
| 40 |  | B013 | Zhuozhou | AMP450 | NW2 | Hull | Fuel |
| 41 |  | C087 | Zhuozhou | AMP450 | NW2 | Hull | Fuel |
| 42 |  | A279 | Jiexiu | AMP450 | NW2 | Hull | Fuel |
| 43 |  | B013 | Jiexiu | AMP450 | NW2 | Hull | Fuel |
| 44 | 10 | A282 | Zhuozhou | 622A | X098 | Hull | Fuel/feed |
| 45 |  | B195 | Zhuozhou | 622A | X098 | Hull | Fuel/feed |
| 46 |  | C278 | Zhuozhou | 622A | X098 | Hull | Fuel/feed |
| 47 |  | A282 | Jiexiu | 622A | X098 | Hull | Fuel/feed |
| 48 |  | C278 | Jiexiu | 622A | X098 | Hull | Fuel/feed |
| 49 | 11 | A027 | Zhuozhou | 624A | X098 | Hull | Fuel |
| 50 |  | B278 | Zhuozhou | 624A | X098 | Hull | Fuel |
| 51 |  | C177 | Zhuozhou | 624A | X098 | Hull | Fuel |
| 52 |  | A027 | Jiexiu | 624A | X098 | Hull | Fuel |
| 53 |  | B278 | Jiexiu | 624A | X098 | Hull | Fuel |
| 54 |  | C177 | Jiexiu | 624A | X098 | Hull | Fuel |
| 55 | 12 | A333 | Zhuozhou | AMP450 | X098 | Hull | Fuel/feed |
| 56 |  | B080 | Zhuozhou | AMP450 | X098 | Hull | Fuel/feed |
| 57 |  | C207 | Zhuozhou | AMP450 | X098 | Hull | Fuel/feed |
| 58 |  | A333 | Jiexiu | AMP450 | X098 | Hull | Fuel/feed |
| 59 |  | B080 | Jiexiu | AMP450 | X098 | Hull | Fuel/feed |
| 60 | 13 | B237 | Zhuozhou | 622A | J7645Z | Hull-less | Food |
| 61 |  | A021 | Jiexiu | 622A | J7645Z | Hull-less | Food |
| 62 |  | B237 | Jiexiu | 622A | J7645Z | Hull-less | Food |
| 63 |  | C062 | Jiexiu | 622A | J7645Z | Hull-less | Food |
| 64 | 14 | A202 | Zhuozhou | 624A | J7645Z | Hull-less | Food |
| 65 |  | C119 | Zhuozhou | 624A | J7645Z | Hull-less | Food |
| 66 |  | A202 | Jiexiu | 624A | J7645Z | Hull-less | Food |
| 67 |  | B206 | Jiexiu | 624A | J7645Z | Hull-less | Food |
| 68 |  | C119 | Jiexiu | 624A | J7645Z | Hull-less | Food |
| 69 | 15 | A252 | Zhuozhou | AMP450 | J7645Z | Hull-less | Feed |
| 70 |  | C066 | Zhuozhou | AMP450 | J7645Z | Hull-less | Feed |
| 71 |  | A252 | Jiexiu | AMP450 | J7645Z | Hull-less | Feed |
| 72 |  | B062 | Jiexiu | AMP450 | J7645Z | Hull-less | Feed |
| 73 |  | C066 | Jiexiu | AMP450 | J7645Z | Hull-less | Feed |
| 74 | 16 | A064 | Zhuozhou | 622A | NW3 | Hull | Fuel |
| 75 |  | B313 | Zhuozhou | 622A | NW3 | Hull | Fuel |
| 76 |  | C222 | Zhuozhou | 622A | NW3 | Hull | Fuel |
| 77 |  | A064 | Jiexiu | 622A | NW3 | Hull | Fuel |
| 78 |  | B313 | Jiexiu | 622A | NW3 | Hull | Fuel |
| 79 |  | C222 | Jiexiu | 622A | NW3 | Hull | Fuel |
| 80 | 17 | A109 | Zhuozhou | 624A | NW3 | Hull | Fuel/feed |
| 81 |  | B164 | Zhuozhou | 624A | NW3 | Hull | Fuel/feed |
| 82 |  | C127 | Zhuozhou | 624A | NW3 | Hull | Fuel/feed |
| 83 |  | A109 | Jiexiu | 624A | NW3 | Hull | Fuel/feed |
| 84 |  | B164 | Jiexiu | 624A | NW3 | Hull | Fuel/feed |
| 85 |  | C127 | Jiexiu | 624A | NW3 | Hull | Fuel/feed |
| 86 | 18 | A150 | Zhuozhou | AMP450 | NW3 | Hull | Fuel/feed |
| 87 |  | B125 | Zhuozhou | AMP450 | NW3 | Hull | Fuel/feed |
| 88 |  | A150 | Jiexiu | AMP450 | NW3 | Hull | Fuel/feed |
| 89 |  | B125 | Jiexiu | AMP450 | NW3 | Hull | Fuel/feed |
| 90 |  | C198 | Jiexiu | AMP450 | NW3 | Hull | Fuel/feed |
| 91 | 19 | A044 | Jiexiu |  | J7645Z | Hull-less | Fuel |
| 92 |  | B033 | Jiexiu |  | J7645Z | Hull-less | Fuel |
| 93 |  | C105 | Jiexiu |  | J7645Z | Hull-less | Fuel |
| 94 | 20 | A139 | Jiexiu | 624A | NW2 | Hull | Fuel |
| 95 |  | B060 | Jiexiu | 624A | NW2 | Hull | Fuel |
| 96 |  | C064 | Jiexiu | 624A | NW2 | Hull | Fuel |
| 97 |  | B060 | Zhuozhou | 624A | NW2 | Hull | Fuel |
| 98 |  | C064 | Zhuozhou | 624A | NW2 | Hull | Fuel |

^a^CMS: Cytoplasmic male-sterile (female); ^b^CMF: Cytoplasmic male-fertile (male)

^c^Samples having more than 15 g·kg^-1^ tannin and 50 g·kg^-1^ hemicellulose content were classified for fuel (Waniska et al., 2015). Feed samples had tannin and hemicellulose content less than 15 g·kg^-1^ and 50 g·kg^-1^ while starch content less than 650 g·kg^-1^ (Waniska et al., 2015). Food samples had starch greater than or equal to 650 g·kg^-1^ used in this study. The tannin and lignocelluloses content in fuel samples was higher than food and feed (Beloshapka et al., 2016; Pontieri et al., 2010; Waniska et al., 2015).

**Figure S1:** Statistical analysis (t-test) between grains and flours to determine the prediction accuracy of models for biochemical components which includes starch, protein, fat, tannin, cellulose, hemicellulose, lignin, and ash. P < 0.05 was used to determine the significant differences between grains and flours models.


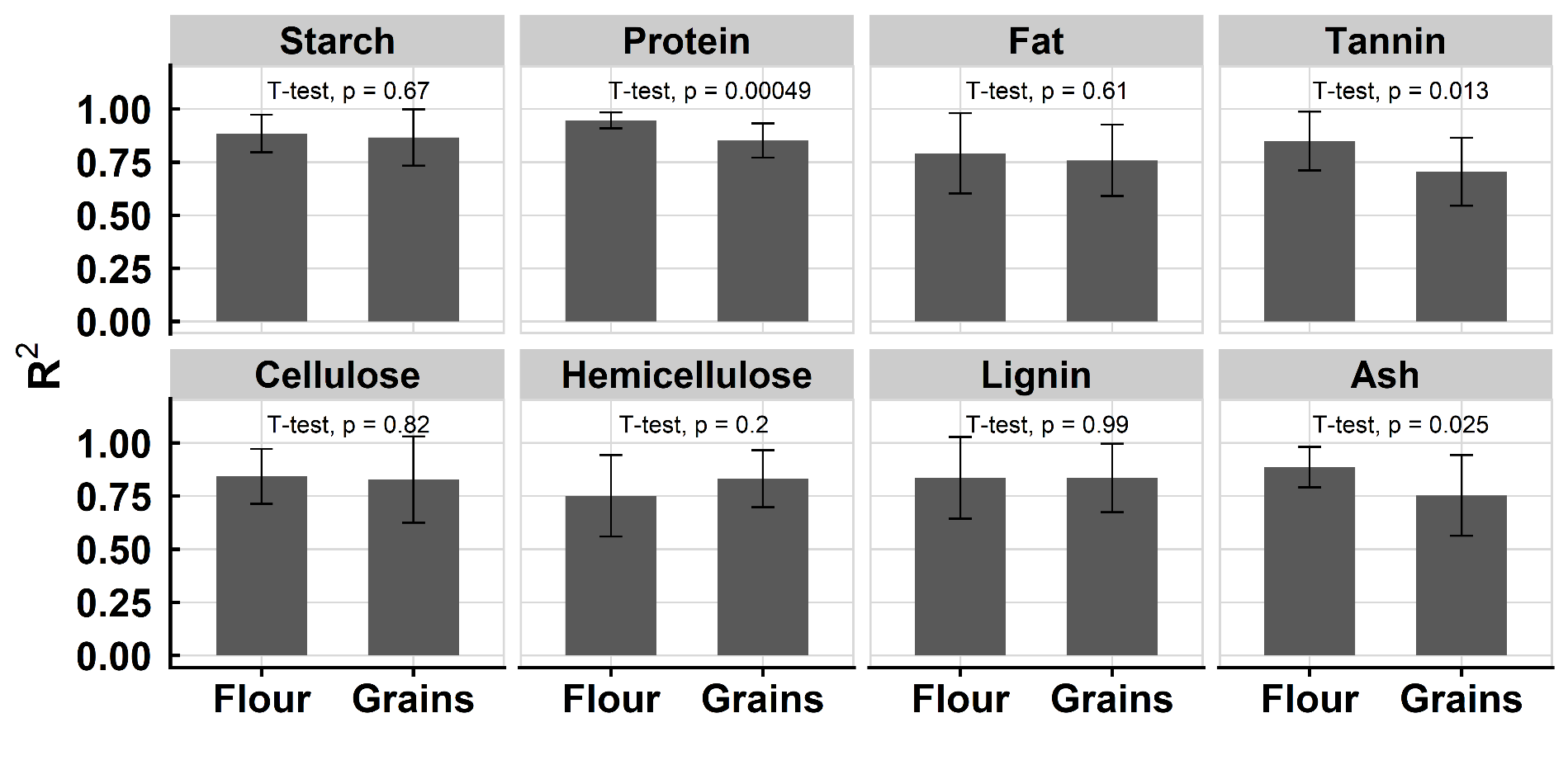


**Figure S2:** Statistical analysis (t-test) between hulled and hull-less grains to determine the prediction accuracy of models for biochemical components which includes starch, protein, fat, tannin, cellulose, hemicellulose, lignin, and ash. P < 0.05 was used to determine the significant differences between grains and flours models.


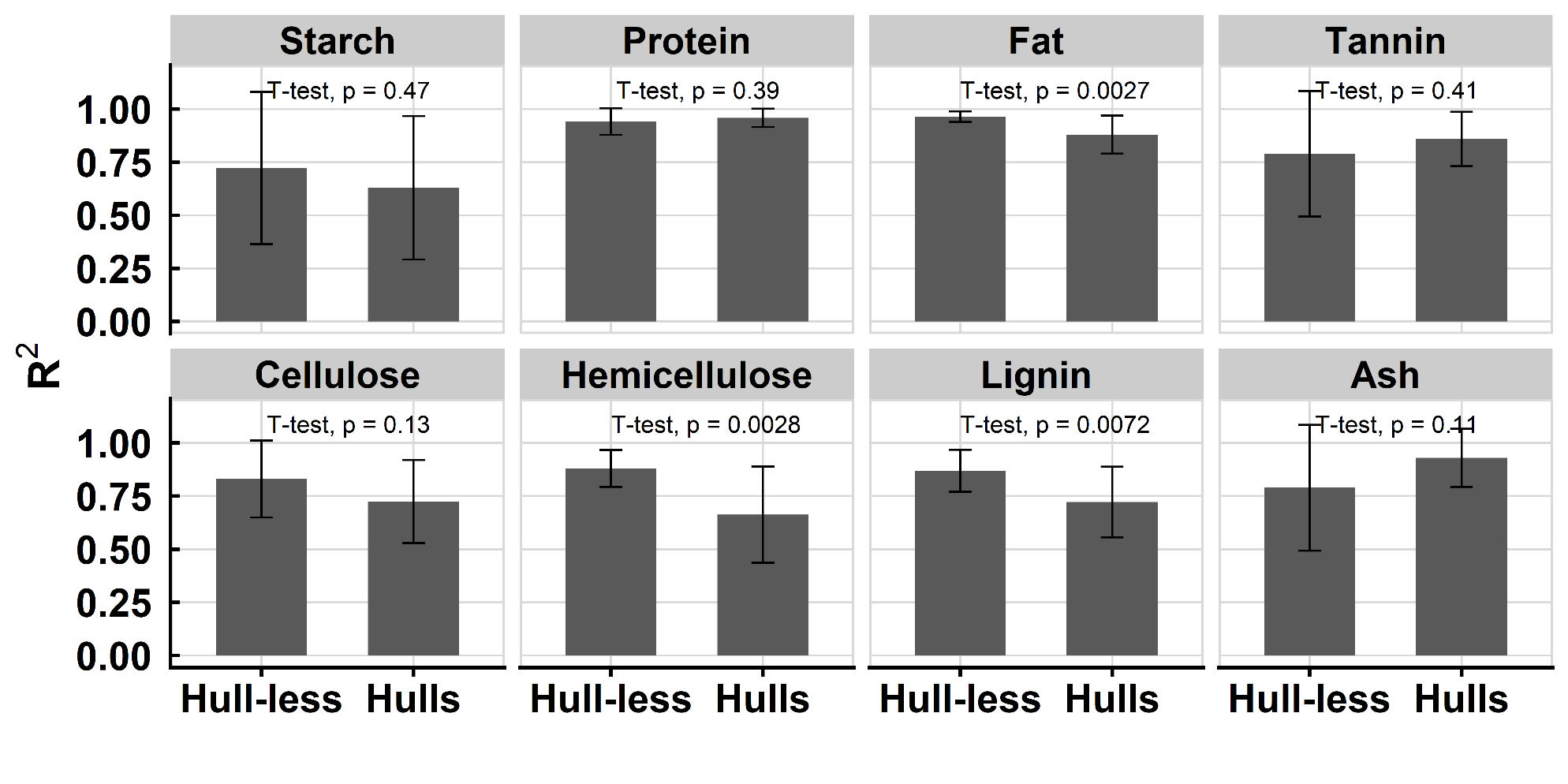

Supplement: Supplementary file 1 [file Data_Sheet_1.docx]
